# Cellular variant focal segmental glomerulosclerosis during trastuzumab emtansine treatment in HER2-positive breast cancer: a case report

**DOI:** 10.3389/fonc.2026.1871262

**Published:** 2026-08-03

**Authors:** Soshi Okada, Sae Aratani, Daisuke Miyamoto, Masujiro Makita, Risa Murasato, Hideko Hoshina, Akio Hirama, Yukako Shintani-Domoto, Tetsuya Kashiwagi, Ryuji Ohashi, Masato Iwabu

**Affiliations:** 1 Department of Endocrinology, Metabolism and Nephrology, Graduate School of Medicine, Nippon Medical School, Tokyo, Japan; 2 Division of Cancer Cell Biology, Institute of Medical Science, University of Tokyo, Tokyo, Japan; 3 Department of Nephrology, Nippon Medical School Musashikosugi Hospital, Kawasaki-shi, Kanagawa, Japan; 4 Department of Breast Surgery and Oncology, Nippon Medical School Musashikosugi Hospital, Kawasaki-shi, Kanagawa, Japan; 5 Department of Diagnostic Pathology, Nippon Medical School, Tokyo, Japan

**Keywords:** case report, focal segmental glomerulosclerosis, HER2-positive breast cancer, onconephrology, trastuzumab emtansine

## Abstract

Onconephrology is an emerging subspecialty that encompasses comprehensive kidney management in patients with cancer. Trastuzumab emtansine (T-DM1) is an antibody–drug conjugate composed of trastuzumab linked to the microtubule inhibitor. T-DM1 is widely used in the treatment of human epidermal growth factor receptor 2-positive breast cancer, and glomerular disease arising during T-DM1 therapy remains rarely reported. The present report describes the case of a 50-year-old Japanese woman who developed nephrotic-range proteinuria approximately five months after initiating T-DM1 adjuvant therapy. Kidney biopsy confirmed the diagnosis of cellular variant focal segmental glomerulosclerosis. Other secondary causes, including autoimmune disease and infection, were excluded, and the temporal relationship between symptom onset and T-DM1 administration suggested a possible drug-related etiology. Given the absence of alternative treatment regimens for her breast cancer, we decided to continue T-DM1 without immunosuppressive therapy, with careful monitoring of kidney function and proteinuria. The scheduled anticancer treatment course was successfully completed, and proteinuria improved significantly within 7 weeks of treatment completion. This case highlights the importance of recognizing rare glomerular adverse effects of T-DM1 and underscores the need to carefully balance oncological treatment benefits against renal toxicity, emphasizing the value of multidisciplinary collaboration between oncologists and nephrologists.

## 1 Introduction

Onconephrology is an emerging subspecialty that integrates nephrology, oncology, and hematology, focusing on the comprehensive management of cancer patients experiencing renal injury. The recognition of its importance has evolved remarkably since its first inception over the past 15 years (1). This evolution can be attributed to the increasing discovery and introduction of novel new anti-cancer drugs. Subsequently, there has been more diverse renal toxicities associated with these therapies.

The clinical scope of onconephrology is broad, encompassing acute kidney injury (AKI), electrolyte disorders, tumor lysis syndrome, direct tumor infiltration to the kidneys, paraproteinemia. Among these, anticancer drug-related nephropathies represent an important and increasingly recognized cause of kidney injury, and may present as AKI, glomerular disease, or electrolyte disturbances, depending on the underlying mechanism. Recently, several molecular-targeted and immune-targeted anticancer therapies have been implicated in the development of nephropathies, including acute tubular injury, acute tubulointerstitial nephritis, renal thrombotic microangiopathy, minimal change disease, and focal segmental glomerulosclerosis (FSGS) (2–4).

Trastuzumab emtansine (T-DM1) is an antibody–drug conjugate composed of trastuzumab linked to the microtubule inhibitor emtansine (DM1), designed to deliver targeted cytotoxicity to HER2-positive cells (5). It has demonstrated efficacy as second-line therapy for locally advanced or metastatic HER2-positive breast cancer (6) and as adjuvant therapy for residual invasive disease following neoadjuvant treatment (7). Commonly reported adverse events include thrombocytopenia, liver dysfunction, peripheral neuropathy, and electrolyte disturbances such as hypokalemia (8). Renal adverse effects, however, remain poorly characterized. Given the rarity of reported cases, the pathological features, management, and prognosis of T-DM1–associated kidney injury remain unclear.

The key questions that need to be discussed among onconephrologists will include whether it is feasible to continue anti-cancer drugs, and if so, how best to support patients through that therapy. Herein, we present a patient with HER2-positive breast cancer who developed the cellular variant of focal segmental glomerulosclerosis during adjuvant T-DM1 therapy. The patient was referred to the department of nephrology for the evaluation of nephrotic-range proteinuria (urine protein-to-creatinine ratio [UPCR], 3.6 g/gCr) without peripheral edema. Despite persistent proteinuria, the patient successfully completed the planned treatment course with preserved kidney function. We describe the pathological findings, clinical course, and long-term outcome of this rare presentation.

## 2 Case presentation

### 2.1 History of present illness

A 50-year-old Japanese woman was referred to the nephrology department for evaluation of persistent proteinuria. She had no history of diabetes mellitus, hypertension, or kidney disease. Her medical history included essential tremors and tension-type headache, treated with arotinolol and alprazolam. One year before presentation, she was diagnosed with human epidermal growth factor receptor 2 (HER2)-positive bilateral invasive ductal carcinoma of the breast, for which she received neoadjuvant chemotherapy with docetaxel (75 mg/m² [107 mg per cycle]), carboplatin (target area under the curve 6 [570 mg per cycle]), and trastuzumab (8 mg/kg loading dose followed by 6 mg/kg) every 3 weeks for six cycles. She subsequently underwent bilateral partial mastectomy and sentinel lymph node biopsy, which revealed residual invasive disease.

**Figure 1:**
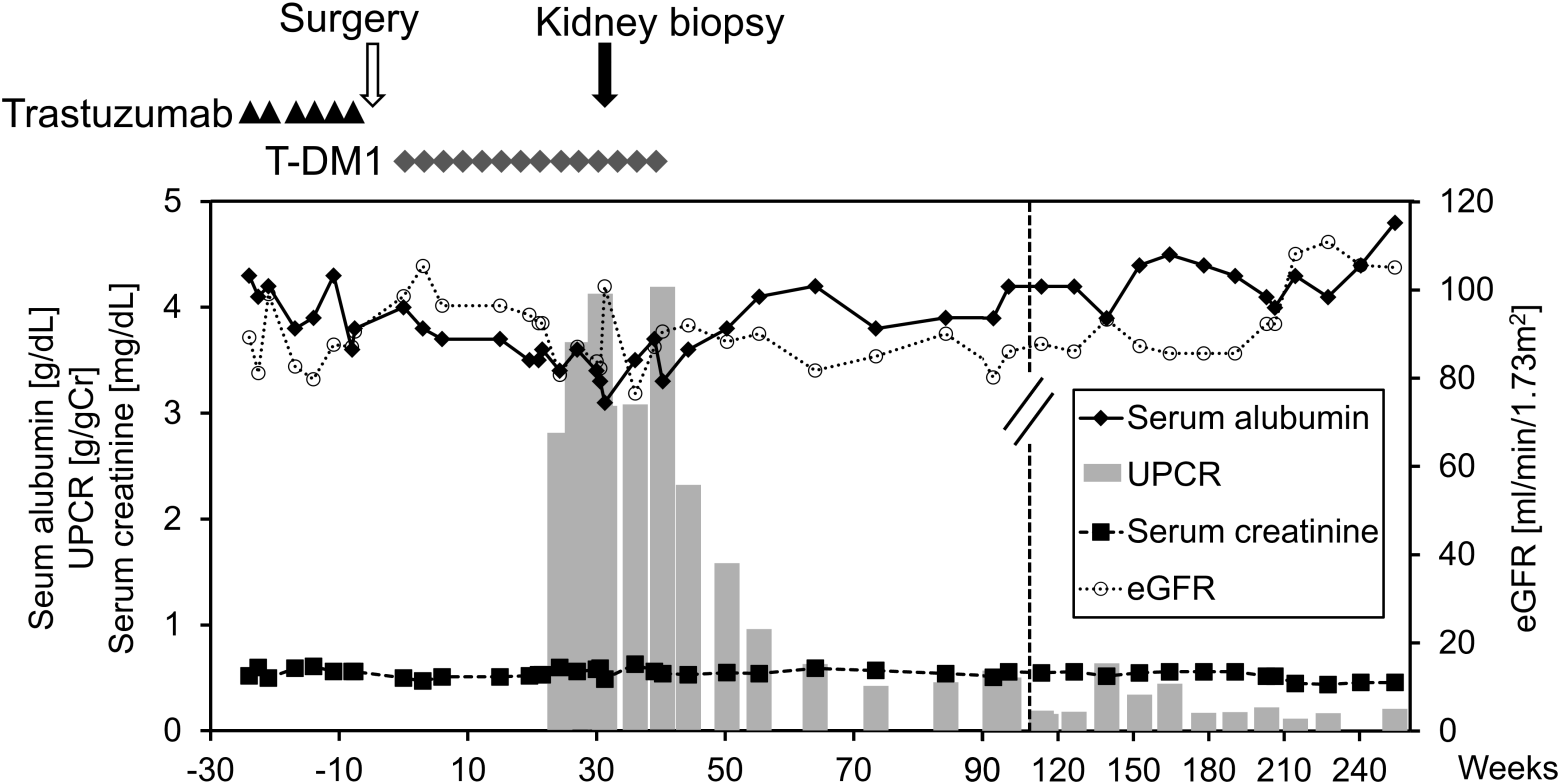
Clinical course of the patient. Changes in serum albumin levels, UPCR, and serum creatinine levels during treatment are shown. T-DM1: trastuzumab emtansine; UPCR: urine protein-to-creatinine ratio.

Adjuvant trastuzumab emtansine (T-DM1) was initiated at 3.6 mg/kg every 3 weeks for a planned total of 14 cycles. Before treatment initiation, kidney function was normal and urinalysis revealed no proteinuria (**Figure 1**). Following the seventh cycle, approximately 5 months after initiation, she reported foamy urine and dipstick urinalysis revealed 3+ proteinuria. Because proteinuria persisted, she was referred for nephrological evaluation.

At presentation, her blood pressure was 119/88 mmHg and her body mass index was 19.7 kg/m². Physical examination revealed no peripheral edema. Laboratory testing revealed a serum total protein 6.6 g/dL, a serum albumin level of 3.4 g/dL, and a serum creatinine level of 0.60 mg/dL (**Table 1**). Urinalysis demonstrated nephrotic-range proteinuria (urine protein-to-creatinine ratio [UPCR], 3.6 g/gCr) without hematuria. Serologic testing for HIV, hepatitis B virus, and hepatitis C virus was negative. Autoimmune serologies, including antinuclear antibody, antineutrophil cytoplasmic antibody, and anti-glomerular basement membrane antibody, were also negative, and complement levels were normal. Both serum and urine immunofixation electrophoresis revealed no monoclonal protein. Kidney ultrasonography demonstrated normal-sized kidneys and computed tomography showed no renal vascular abnormality. A kidney biopsy was subsequently performed to determine the etiology of nephrotic-range proteinuria.

**Table 1:** Laboratory analysis on admission.

Parameter	Value		Reference range
WBC	9,110	/µL	3,300–8,600
Hb	12.6	g/dL	11.6–14.8
PLT	24.2	×10^4^/µL	15.8–34.8
TP	6.6	g/dL	6.6–8.1
Alb	3.4	g/dL	4.1–5.1
BUN	17.5	mg/dL	8–20
Cr	0.6	mg/dL	0.46–0.79
Na	142	mEq/L	138–145
K	3.3	mEq/L	3.6–4.8
Mg	1.5	mg/dL	1.8–2.4
LDL-C	180	mg/dL	< 120
Glu	113	mg/dL	70–109
HbA1c	5.9	%	≤ 6.0
IgG	1,246	mg/dL	861–1,747
IgA	239	mg/dL	93–393
IgM	209	mg/dL	50–269
C3	148	mg/dL	73–138
C4	19.7	mg/dL	11–31
CH50	54	U/mL	32–44
ANA	< 40		< 40
uPCR	2.81	g/gCr	
U-B2MG	180	µg/L	≤ 230
U-RBC	1–4	/HPF	
Hyaline casts	20–29	/WF	

Alb, albumin; ANA, antinuclear antibody; BUN, blood urea nitrogen; CH50, 50% hemolytic complement activity; C3, complement component 3; C4, complement component 4; Cr, creatinine; Glu, glucose; Hb, hemoglobin; HbA1c, hemoglobin A1c; HPF, high power field; IgA, immunoglobulin A; IgG, immunoglobulin G; IgM, immunoglobulin M; K, potassium; LDL-C, low-density lipoprotein cholesterol; Mg, magnesium; Na, sodium; PLT, platelets; TP, total protein; U-B2MG, urinary β2-microglobulin; UPCR, urine protein-to-creatinine ratio; U-RBC, urinary red blood cells; WBC, white blood cell; WF, whole field.

### 2.2 Pathological findings

**Figure 2:**
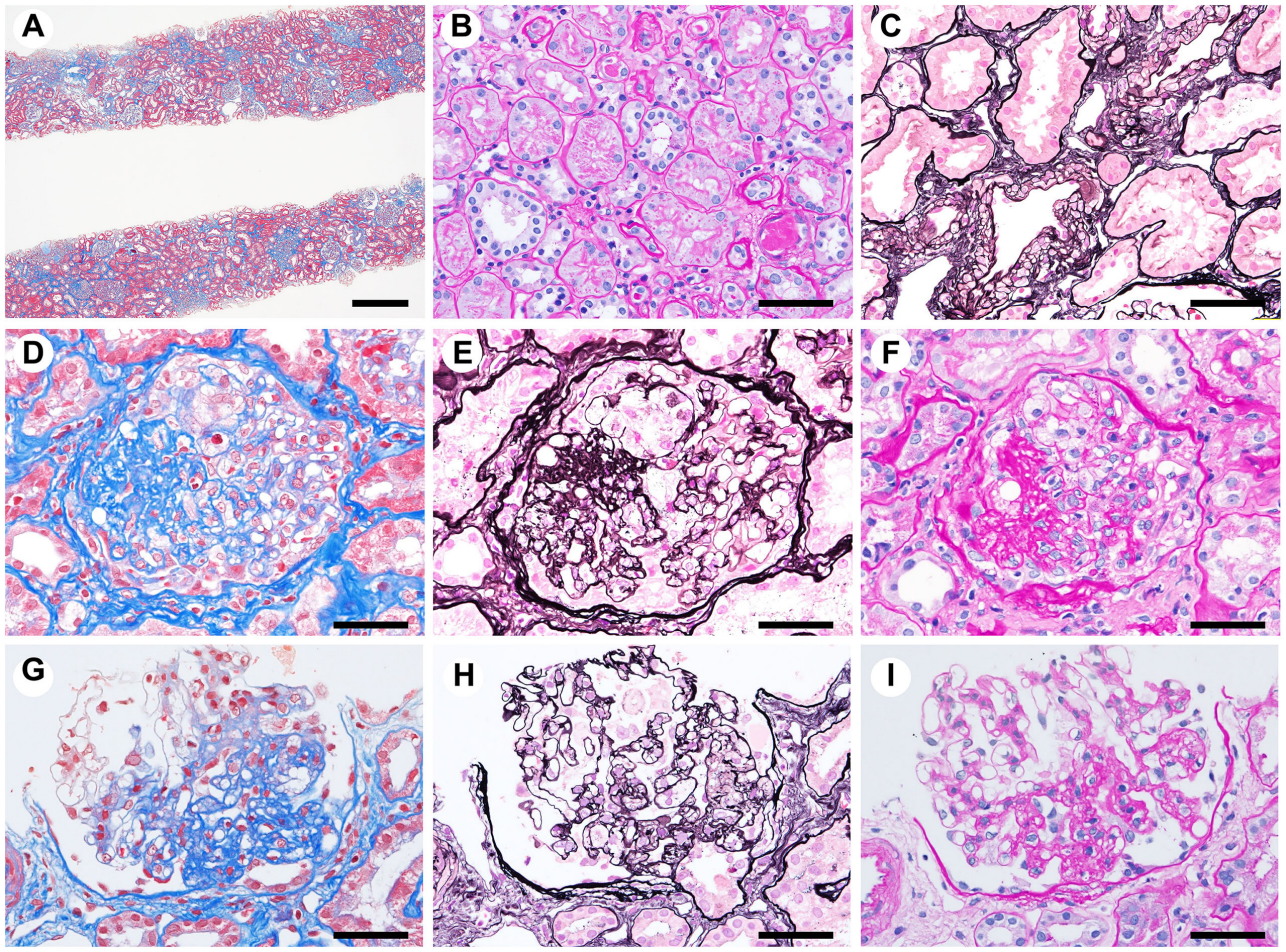
Pathological findings of kidney biopsy on light microscopy. **(A)** Low-magnification of a kidney biopsy specimen stained with Masson’s trichrome, depicting interstitial fibrosis involving approximately 10–20% of the cortex. **(B)** Proximal tubules containing numerous reabsorption droplets, highlighted by PAS staining. **(C)** Mild-to-moderate arteriosclerosis demonstrated by silver staining. **(D–I)** Masson’s trichrome staining **(G, J)**, silver staining **(H, K)**, and PAS staining **(I, L)**. Two glomeruli exhibited segmental endocapillary hypercellularity, with the involved segment expanded by endocapillary cells predominantly comprising foam cells. Segmental sclerosis is also present. Scale bars: 500 μm **(A)**; 50 μm **(B–L)**. PAS, periodic acid–Schiff.

**Figure 3:**
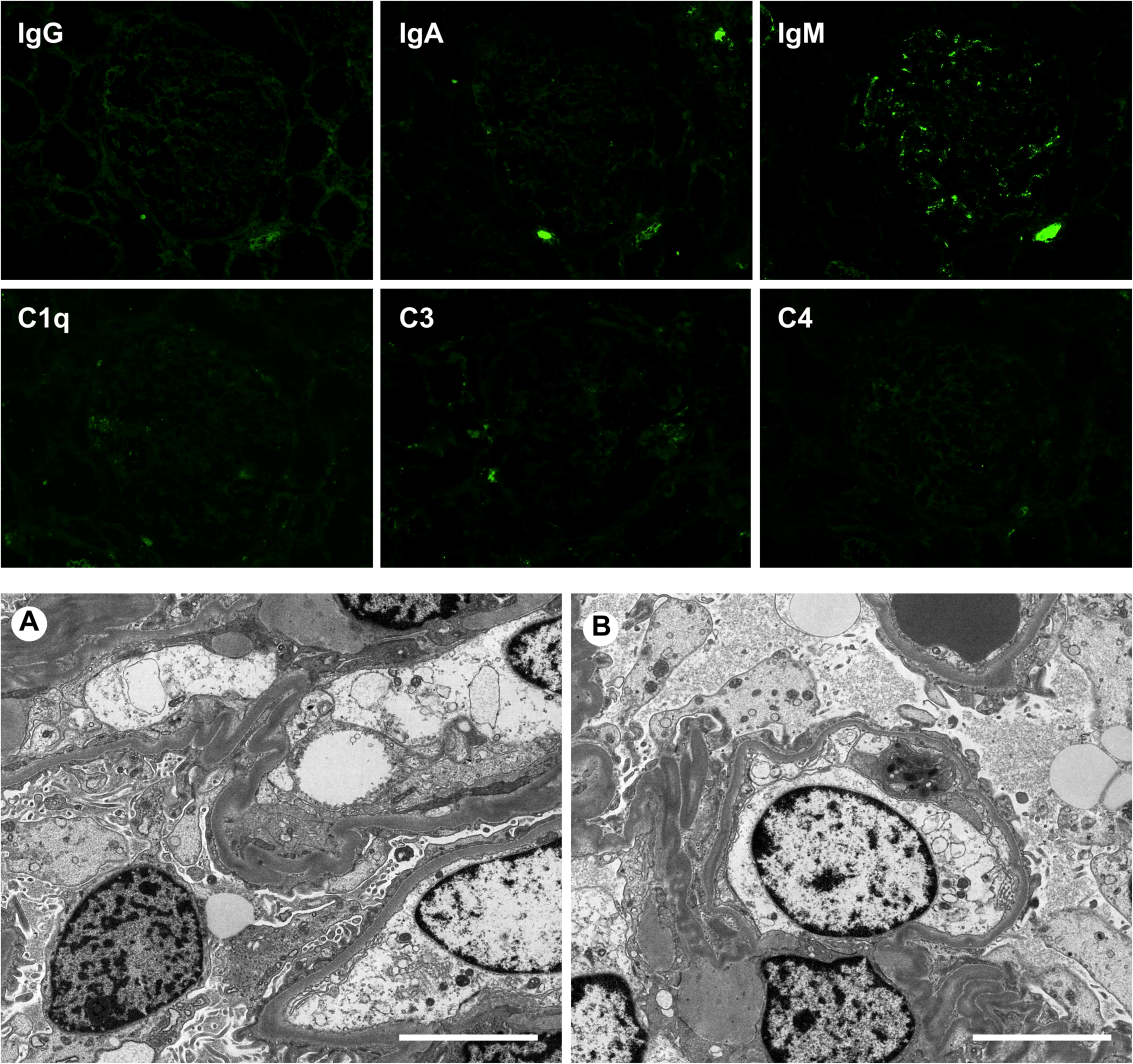
Immunofluorescence microscopy and electron microscopy. Immunofluorescence showed trace IgM staining; IgG, IgA, C1q, C3, and C4 were all negative. Electron microscopy demonstrating segmental foot process effacement without electron-dense deposits. C1q, complement component 1q; C3, complement component 3; C4, complement component 4; IgA, immunoglobulin A; IgG, immunoglobulin G; IgM, immunoglobulin M Original magnification ×200. **(A, B)** Electron microscopy demonstrated segmental enlargement of podocyte nuclei with foot process effacement. Scale bars: 5 μm.

The biopsy specimen contained 35 glomeruli for light microscopic analysis (**Figure 2**). Two glomeruli exhibited segmental endocapillary hypercellularity with expansion and occlusion of the capillary lumina, composed predominantly of foam cells, accompanied by segmental sclerosis. No glomerular tuft collapse with overlying podocyte hypertrophy or hyperplasia was identified, and the segmental lesions were not confined to the proximal tubular pole. Additionally, three obsolescent glomeruli with ischemic changes were noted. Tubulointerstitial fibrosis with inflammatory cell infiltration involved approximately 10–20% of the cortical area, and mild-to-moderate arteriosclerosis was present. There were no features of thrombotic microangiopathy, such as glomerular double contours or microthrombi. Immunofluorescence microscopy revealed trace immunoglobulin M staining, with negative staining for immunoglobulin G, immunoglobulin A, complement component 3, and complement component 1q (**Figure 3**). Electron microscopy demonstrated segmental, rather than diffuse, foot process effacement without electron-dense deposits (**Figure 3**). Collectively, these findings supported an FSGS diagnosis, cellular variant.

### 2.3 Clinical course

Established causes of secondary FSGS, including infectious and autoimmune diseases, obesity, reduced renal mass, and medication-related factors other than T-DM1, were systematically excluded. Given the temporal occurrence between T-DM1 initiation and the onset of nephrotic-range proteinuria, a drug-related secondary FSGS was considered the most likely explanation. The clinical course is summarized in **Figure 1**.

The decision to continue T-DM1 was reached through a multidisciplinary discussion involving the departments of breast oncology and nephrology. As adjuvant T-DM1 was considered essential for improving invasive disease-free survival in the present patient with residual invasive disease (7) and no alternative regimen of comparable efficacy was available, the anticipated oncological benefit was deemed to outweigh the risk of progressive kidney injury, particularly given the preserved kidney function and absence of overt nephrotic syndrome. Following a thorough explanation of the potential risks, including progression of proteinuria and kidney injury, the patient consented to continue treatment under close surveillance. Immunosuppressive therapy was not initiated, as the lesion was considered a drug-related secondary podocytopathy. Supportive nephrological management consisted of losartan 25 mg/day, with kidney function, proteinuria, and blood pressure monitored every 3 weeks throughout the remaining treatment course.

During the remainder of therapy, the serum albumin level of the patient remained above 3.0 g/dL, proteinuria persisted with a UPCR of approximately 3–4 g/gCr, the estimated glomerular filtration rate (eGFR) remained stable at 80–100 mL/min/1.73 m², and the systolic blood pressure was 100–110 mmHg. The patient completed the planned adjuvant T-DM1 course. Following treatment completion, proteinuria gradually decreased and the UPCR improved to 0.9 g/gCr 7 weeks after the final dose. Losartan was discontinued 1 year later, owing to low blood pressure. At the most recent follow-up, approximately 3.5 years after completion of T-DM1, the eGFR of the patient was 105 mL/min/1.73 m², the UPCR improved further to 0.21 g/gCr, and the blood pressure was 137/80 mmHg, with no breast cancer recurrence noted. The patient was satisfied to complete the planned adjuvant therapy while maintaining stable kidney function under close nephrological follow-up.

## 3 Discussion

In this report, we present a case of cellular variant FSGS that developed during T-DM1 therapy. The subsequent improvement of proteinuria following treatment completion suggests a potential relationship between T-DM1 and FSGS development. Notably, the decision to continue T-DM1 treatment was made in the absence of an alternative regimen of comparable efficacy being available and as it was deemed essential for improving invasive disease-free survival (7). Despite the presence of nephrotic-range proteinuria, our patient did not develop nephrotic syndrome or acute kidney injury. Ultimately, she completed the planned course of adjuvant T-DM1 therapy with preservation of kidney function throughout the treatment period. Furthermore, no recurrence of breast cancer was observed at the most recent follow-up.

In the present case, histological classification was based on the Columbia working classification of FSGS (9). The defining feature of the cellular variant is segmental endocapillary hypercellularity occluding the capillary lumina, frequently accompanied by foam cells and infiltrating leukocytes. These findings occur in the absence of the features defining the collapsing and tip variants, which take diagnostic precedence. In our case, the absence of segmental or global tuft collapse with overlying podocyte hyperplasia excluded the collapsing variant, while the absence of a lesion restricted to the tubular pole excluded the tip variant. Therefore, the findings were best categorized as the cellular variant. Moreover, the essentially negative immunofluorescence and absence of electron-dense deposits excluded an immune complex–mediated proliferative glomerulonephritis. Because the cellular variant is defined by light-microscopic criteria under the Columbia classification, whereas the extent of foot process effacement is an ultrastructural feature distinguishing primary from secondary FSGS, the segmental (rather than diffuse) effacement observed here is compatible with the cellular variant and more consistent with secondary rather than primary FSGS, in which the effacement is typically diffuse (10). Moreover, we identified no recognized cause of secondary FSGS, including obesity, a marked reduction in nephron number, use of certain drugs (bisphosphonates, interferon, calcineurin inhibitors, or mechanistic target of rapamycin [mTOR] inhibitors), or viral infection. The globally obsolescent glomeruli numbered only 3 of the 35 sampled, which is within the range expected for the age of the patient (11). Furthermore, the observed ischemic, obsolescent morphology was consistent with age-related rather than disease-related glomerulosclerosis. The mild tubulointerstitial fibrosis was accompanied by only sparse, nonspecific inflammatory infiltration without prominent tubulitis or eosinophils, most consistent with a secondary, reactive change accompanying the globally obsolescent glomeruli and arteriosclerosis rather than a primary interstitial process; consistent with this, urinary β2-microglobulin, a marker of proximal tubular and tubulointerstitial injury, was within the normal range.

FSGS is a renal histologic pattern of injury arising from primary or secondary causes, ultimately resulting in segmental glomerulosclerosis (10, 12). The Kidney Disease: Improving Global Outcomes 2021 guidelines propose an etiology-based classification of FSGS into primary, genetic, secondary, and undetermined causes (13). Secondary FSGS is associated with viral infections, maladaptive hemodynamic responses, obesity-related factors, primary glomerular diseases, systemic conditions, and drug exposure. Drugs historically implicated in FSGS include interferons, bisphosphonates, lithium, sirolimus, antiviral therapeutics, and anthracyclines (12, 14, 15). More recently, molecular-targeted therapies and immune-regulated therapies have been recognized as potential contributors to the development of FSGS (2, 16). FSGS results from podocyte injury and subsequent glomerulosclerosis, ultimately leading to kidney dysfunction (9). Drug-induced podocytopathy can arise through various mechanisms, including direct podocyte toxicity, disruption of podocyte–endothelial signaling, and immune-mediated injury (17, 18).

In the present case, before adjuvant T-DM1, our patient had received neoadjuvant docetaxel, carboplatin, and trastuzumab. However, several lines of evidence argue against a primary contribution of these agents to the observed lesion. First, urinalysis revealed no proteinuria before the T-DM1 initiation, after treatment with these agents had been completed. Moreover, nephrotic-range proteinuria emerged only during T-DM1 therapy (**Figure 1**), temporally dissociating the prior regimen from the onset of the podocytopathy. Second, platinum agents typically produce tubular injury rather than a podocytopathy (19) and taxanes and trastuzumab as single agents are not currently considered nephrotoxic (20, 21). Consistent with this interpretation, the only previously reported case of FSGS during T-DM1 therapy followed a different neoadjuvant regimen, comprising a taxane with pertuzumab and trastuzumab, without a platinum agent (22). Notably, the FSGS lesion similarly emerged only after the introduction of T-DM1, further arguing against the prior cytotoxic agents as the primary cause. Nevertheless, a cumulative or sensitizing effect of prior cytotoxic exposure on podocyte vulnerability cannot be entirely excluded, representing a factor that limits causal attribution to T-DM1 in a single case.

Beyond the prior chemotherapy, we also considered a paraneoplastic FSGS arising from the underlying malignancy itself. Although paraneoplastic glomerulopathy characteristically tracks with tumor activity (23), no proteinuria was detected before or after neoadjuvant chemotherapy; nephrotic-range proteinuria emerged only during T-DM1 therapy after resection of the primary tumor and improved after T-DM1 completion. Moreover, computed tomography during her evaluation revealed no other malignancy. This course indicates linkage to T-DM1 exposure rather than tumor activity, rendering a paraneoplastic FSGS unlikely.

The pathogenesis underlying podocyte injury that may occur during T-DM1 therapy remains unknown. As T-DM1 is an antibody–drug conjugate, it could theoretically affect podocytes through two distinct mechanisms. First, a payload-dependent, antigen-independent effect does not require HER2 binding or internalization. In particular, the DM1 moiety can directly bind cytoskeleton-associated protein 5 (CKAP5) at the cell surface, triggering plasma-membrane damage, calcium influx, and secondary microtubule disorganization in cells with low or absent HER2 expression (24). Given the wide expression of CKAP5 across human tissues, an analogous surface-mediated, payload-driven effect on podocytes is biologically plausible; however, this mechanism has been demonstrated in hepatocytes rather than podocytes and therefore remains speculative in the present context. Second, an antibody-mediated, HER2-dependent mechanism also warrants consideration. This includes not only internalization with intracellular payload release, but also Fc-mediated effector functions such as antibody-dependent cellular cytotoxicity (ADCC), which T-DM1 retains from trastuzumab (5). Both routes require binding of trastuzumab to HER2 on the target podocyte. However, HER2 expression in the human kidney has been documented mainly in proximal tubular epithelial cells, whereas podocyte expression is not well established (25), rendering both routes unlikely to predominate in podocytes. Taken together, although a direct payload-mediated effect, HER2-dependent internalization, and Fc-mediated ADCC are each biologically conceivable, none can be firmly established in the absence of functional or molecular confirmation, and the precise mechanism underlying T-DM1–associated podocyte injury therefore remains undetermined.

To date, only one case of acute kidney injury and proteinuria following T-DM1 therapy has been reported, in which a kidney biopsy revealed collapsing FSGS with tubular injury (22). Notably, the patient had pre-existing type 2 diabetes with established diabetic nephropathy and presented with a hypertensive crisis and acute kidney injury. In contrast, our patient had no underlying kidney disease and demonstrated a cellular variant with preserved kidney function. This contrast underscores the histological and clinical heterogeneity of T-DM1-associated glomerular injury and suggests that the resulting phenotype may be modulated by individual susceptibility. However, in both cases, proteinuria improved after discontinuation of T-DM1 without specific immunosuppressive therapy, with kidney function also recovering in the previously reported patient. This response to drug withdrawal supports a drug-related etiology. T-DM1 is mainly eliminated through bile following conversion to DM1-containing catabolites, with minimal renal elimination, which may explain the relatively low frequency of acute kidney injury in patients receiving this agent. Nonetheless, persistent proteinuria is an independent and significant risk factor for the development of chronic kidney disease, underscoring the importance of careful observation.

This report has certain limitations. First, it involved only a single case. Second, a contribution from the prior chemotherapeutic agents to the podocytopathy cannot be entirely excluded. Third, we did not perform drug rechallenge. Finally, we did not conduct functional or molecular studies to confirm the mechanism of injury. Collectively, these factors preclude a definitive causal attribution; any mechanistic interpretation remains hypothetical, and the association should be interpreted as temporal and probable rather than established.

In conclusion, the present report described a cellular variant of FSGS that developed during T-DM1 therapy and was accompanied by transient nephrotic-range proteinuria, suggesting a possible drug-related association. However, the therapeutic benefits of T-DM1 outweighed this potential renal adverse effect in this case and the patient successfully completed the scheduled chemotherapy regimen with preserved kidney function.

## Data Availability

The original contributions presented in the study are included in the article/supplementary material. Further inquiries can be directed to the corresponding authors.
